# Subclavian artery stenosis in hemodialysis patients: a case series and review of the literature

**DOI:** 10.3389/fphar.2025.1689306

**Published:** 2026-01-09

**Authors:** Wenshen Pu, Jingjie Yang, Qiquan Lai, Yu Zhou, Bo Tu, Bingjie Wang, Ziming Wan

**Affiliations:** 1 Department of Nephrology, Baoshan People’s Hospital, Baoshan, China; 2 Department of Radiology, The First Affiliated Hospital of Chongqing Medical University, Chongqing, China; 3 Department of Nephrology, The First Affiliated Hospital of Chongqing Medical University, Chongqing, China; 4 Department of Ultrasonography, The First Affiliated Hospital of Chongqing Medical University, Chongqing, China

**Keywords:** arteriovenous fistula dysfunction, hemodialysis, percutaneous transluminal angioplasty, subclavian artery stenosis, ultrasound

## Abstract

Subclavian artery (SA) stenosis is an uncommon but important cause of arteriovenous fistula (AVF) dysfunction in hemodialysis patients, yet its diagnosis is frequently delayed because symptoms are nonspecific and often attributed to venous lesions. We report a case series of nine hemodialysis patients diagnosed with SA stenosis or occlusion at a tertiary nephrology center between 2019 and 2023, detailing their clinical presentations, imaging findings, treatments, and outcomes, and summarize relevant literature to contextualize our findings. All patients had left-sided AVFs, mostly radial-cephalic, with a median interval of 3.4 years from dialysis initiation to diagnosis. Presentations included reduced AVF blood flow, occlusion, diminished thrill, and significant interarm systolic blood pressure discrepancy, two patients reported neurological symptoms. Duplex ultrasound often revealed reduced brachial artery flow and/or reversed vertebral artery flow, while lesions were most frequently located at the SA origin. Atherosclerosis was the predominant etiology, followed by Takayasu arteritis. Seven patients underwent percutaneous transluminal angioplasty (PTA), six with stenting, achieving restoration of AVF flow and symptom resolution, two patients declined intervention but maintained adequate dialysis. Literature review confirmed that high-flow AVFs and atherosclerosis increase the risk of SA stenosis and that endovascular revascularization is the preferred treatment when access dysfunction is present. Our findings highlight the importance of early screening with bilateral blood pressure measurement and duplex ultrasound in patients with unexplained access dysfunction, and we outline a practical diagnostic and treatment approach that may help improve dialysis adequacy and reduce morbidity in this high-risk population.

## Introduction

Arteriovenous fistula (AVF) remains the preferred vascular access for maintenance hemodialysis due to its superior long-term patency and lower complication rates compared with arteriovenous grafts and central venous catheters (Lok et al., 2020). However, AVF dysfunction is a common and significant clinical challenge that compromises dialysis adequacy and patient outcomes. The causes of AVF dysfunction are multifactorial and include outflow stenosis, inflow stenosis, thrombosis, and central venous stenosis (Duijm et al., 2006; Wu et al., 2012). While outflow lesions have traditionally received the most attention, increasing evidence underscores the importance of arterial inflow pathology, which has been reported in up to 35% of dysfunctional accesses (Caeiro et al., 2013). Among the etiologies of inflow stenosis, subclavian artery (SA) stenosis is relatively uncommon but clinically significant. SA stenosis may result in hemodynamic steal phenomena, reduced AVF blood flow (Kargiotis et al., 2016).

The prevalence of SA stenosis is approximately 2%–7% in the general or clinical population, with higher rates reported in patients with peripheral arterial disease, diabetes, hypertension, and a history of tobacco use (Shadman et al., 2004). Hemodialysis patients are at even greater risk due to their increased burden of systemic atherosclerosis and chronic inflammatory status (O'Har et al., 2002; Raikou and Kyriaki, 2019). However, the diagnosis of SA stenosis in hemodialysis patients is often delayed or overlooked due to nonspecific symptoms and the tendency to attribute AVF dysfunction to venous abnormalities alone. Prior studies have described isolated cases of subclavian steal syndrome or coronary-subclavian steal in dialysis patients, but systematic descriptions of SA stenosis manifesting as access dysfunction remain limited, and most published evidence consists of single-case reports or small observational cohorts (Kuo et al., 2010; Tan et al., 2013; Alemzadeh-Ansari et al., 2021). In clinical practice, this condition may be underdiagnosed unless physicians actively assess blood pressure discrepancies between arms, thrill or bruit changes, or reversed vertebral artery flow on duplex ultrasonography (Kalaria et al., 2005).

In this study, we present a case series of nine hemodialysis patients diagnosed with SA stenosis or occlusion at a nephrology institution of a tertiary hospital between 2019 and 2023. We detail their clinical features, imaging findings, and treatment outcomes. Furthermore, we review the available literature to summarize the treatment strategies of SA stenosis in the context of dialysis vascular access.

### Case presentation

Between 2019 and 2023, 4514 hemodialysis patients presenting with AVF dysfunction were admitted at a Chongqing tertiary hospital. During this period, SA stenosis or occlusion was identified in 9 patients (four women), and all of these cases were consecutively included in this case series, yielding a crude incidence of 0.2% (9/4514). The degree of SA stenosis was classified according to the North American Symptomatic Carotid Endarterectomy Trial (NASCET) criteria as normal, 50%–69% (stenosis), and 70%–99% (severe stenosis). Patient characteristics are summarized in Table 1. The median age at diagnosis was 67 years (range, 51–91). Among them, eight patients had hypertension, three had diabetes mellitus, and five had a history of smoking. The underlying causes of end-stage renal disease (ESRD) included diabetic nephropathy (patients 2, 6, and 9), chronic glomerulonephritis (patients 3 and 5), hypertensive nephropathy (patient 1), kidney stones (patient 4), and anti-neutrophil cytoplasmic antibody-associated vasculitis (patient 7). The cause of ESRD was unknown in one patient (patient 8). The median duration from the initiation of maintenance hemodialysis to the diagnosis of SA stenosis or occlusion was 3.4 years (range, 0.8–7 years). The initial clinical presentations included decreased AVF blood flow observed on dialysis (patients 1–3), AVF occlusion (patients 4 and 7), diminished AVF thrill on examination (patients 5 and 6), and significant interarm systolic blood pressure discrepancy (patients 8 and 9). In addition, patient 1 and 2 reported subjective symptoms of dizziness, and patient 5 experienced numbness in the left upper limb. The remaining patients were asymptomatic.

**TABLE 1:** Patient characteristics, examination and treatment.

Patients	Patient 1	Patient 2	Patient 3	Patient 4	Patient 5	Patient 6	Patient 7	Patient 8	Patient 9
Demographics and medical history									
Sex	M	F	F	F	M	M	F	M	M
Age, y	91	79	51	61	73	65	67	56	63
Hypertension	Yes	Yes	No	Yes	Yes	Yes	Yes	Yes	Yes
Diabetes mellitus	No	Yes	No	No	No	Yes	No	No	Yes
Smoking history	Yes	No	No	No	Yes	Yes	No	Yes	Yes
Causes of ESRD	Hypertensive nephropathy	DN	CGN	Kidney stone	CGN	DN	AAV	NA	DN
Years of HD	7	3	6	0.8	3	1	4	3	3
Chief complaint	ABF declined	ABF declined	ABF declined	AVF occlusion	AVF thrill weak	AVF thrill weak	AVF occlusion	Unequal blood pressure of both upper limbs	Unequal blood pressure of both upper limbs
Combined symptoms	Dizzy	Dizzy	Asymptomatic	Asymptomatic	Numbness of left upper limb	Asymptomatic	Asymptomatic	Asymptomatic	Asymptomatic
HD AVF									
Position	Left wrist	Left wrist	Left wrist	Left wrist	Left wrist	Left wrist	Left wrist	Left forearm	Left snuff pit
Fistula	Radial-cephalic	Radial-cephalic	Radial-cephalic	Ulnar-basilic	Radial-cephalic	Radial-cephalic	Radial-cephalic	Radial-cephalic	Radial-cephalic
Years of usage	7	3	6	0.6	3	1	4	3	3
PTA times	2	4	2	0	2	0	4	0	1
Physical examination of AVF									
Brachial artery pulse	Weak	Weak	Weak	Weak	Weak	Weak	Weak	Obvious	Obvious
AVF thrill	Discontinuous	Discontinuous	Discontinuous	None	Discontinuous	Discontinuous	None	Continuous	Continuous
Pulse augmentation test	Weak response	Weak response	Weak response	Negative	Weak response	Weak response	Negative	Normal	Normal
Blood pressure (mmHg)									
AVF arm	107/51	138/61	87/60	178/111	119/66	142/93	107/80	124/69	122/71
Non-AVF arm	132/54	188/72	120/70	193/116	179/76	169/83	138/69	155/75	178/75
AVF DU before treatment									
Blood flow (mL/min)	780	217	191	47	653	174	31	609	548
RI	0.43	0.84	0.33	1	0.42	0.67	1	0.38	0.39
Stenosis or occlusion	Radial artery	Cephalic vein	Cephalic vein	Cephalic vein	No	Cephalic vein	Cephalic vein	No	No
AVF ipsilateral vertebral artery ultrasound	Complete reversed flow	NE	Complete reversed flow	Systole reversed flow	NE	NE	Complete reversed flow	NE	NE
Left SA stenosis or occlusion									
Causes	Atherosclerosis	Takayasu arteritis	Atherosclerosis	Atherosclerosis	Atherosclerosis	Atherosclerosis	Atherosclerosis	Atherosclerosis	Takayasu arteritis
Location of lesion	Origin	Origin	Origin	Proximal	Proximal	Origin	Proximal	Origin	Origin
CTA	Severe stenosis	Severe stenosis	Severe stenosis	Severe stenosis	Severe stenosis	Severe stenosis	Severe stenosis	Severe stenosis	Severe stenosis
DSA	Occlusion	Severe stenosis	Occlusion	Severe stenosis	Severe stenosis	Severe stenosis	Occlusion	NE	NE
Revascularization therapy									
AVF stenosis or occlusion	PTA (Conquest 7 mm × 40 mm)	PTA (Conquest 6 mm × 40 mm)	PTA (Mustang 7 mm × 40 mm)	PTA (Mustang 5 mm × 40 mm, 6 mm × 40 mm)	No lesions	PTA (Mustang 5 mm × 40 mm)	PTA (Mustang 6 mm × 40 mm)	No lesions	No lesions
SA stenosis or occlusion	PTA (RIVAL 5 mm × 40 mm) + stenting (Everflex 6 mm × 40 mm)	PTA (Invatec 5 mm × 50 mm、6 mm × 40 mm) + stenting (Biotronik 7 mm × 40 mm)	PTA (Mustang 6 mm × 40 mm, 7 mm × 40 mm) + stenting (Everflex 7 mm × 40 mm)	PTA (Mustang 5 mm × 40 mm, 7 mm × 40 mm) + stenting (PRECISE PRO 8 mm × 40 mm)	PTA + stenting (Lifestream 6 mm × 37 mm)	PTA (Mustang 5 mm × 40 mm) + stenting (Everflex 8 mm × 40 mm)	PTA (Mustang 5 mm × 40 mm, 7 mm × 40 mm, 8 mm × 40 mm)	NT	NT
Outcome Symptom Dialysis	Remission Normal	Remission Normal	Asymptomatic Normal	Asymptomatic Normal	Not remission Normal	Asymptomatic Normal	Asymptomatic Normal	Asymptomatic Normal	Asymptomatic Normal
AVF DU after treatment									
Blood flow (mL/min)	1054	881	1343	1336	NE	1168	1200	NE	NE
RI	0.60	0.61	0.46	0.5	NE	0.46	0.34	NE	NE
Follow-up									
Primary patency duration (Months)	3.2	5.2	17.8	34.1	Lost follow-up	5.0	4.7	NA	NA
Second patency duration (Months)	3.2	9.9	17.8	34.1	​	16.3	8.3	​	​
Subsequent dialysis access	Right antecubital AVF	Left forearm AVF	Right internal jugular TDC	Patent	​	Right forearm AVG	Dead	​	​

Abbreviations: AAV, antineutrophil cytoplasmic antibody-associated vasculitis; ABF, AVF, blood flow; AVF, arteriovenous fistula; AVG, arteriovenous graft; CGN, chronic glomerulonephritis; cm, centimeter; CTA, computed tomography angiography; DN, diabetic nephropathy; DSA, digital subtraction angiography; DU, duplex ultrasound; ESRD, end-stage renal disease; F, female; HD, hemodialysis; M, male; NA, not applicable; NE, no examination; NT, no treatment; PTA, percutaneous transluminal angioplasty; RI, resistive index; SA, subclavian artery; TDC, tunneled dialysis catheters; y, year.

All patients used left-sided AVFs for hemodialysis. The fistulas were located at the wrist in seven patients and at the anatomical snuffbox or forearm in the remaining two. Eight patients had radial-cephalic fistulas, and one had an ulnar-basilic fistula. Six of the nine patients had previously undergone one or more sessions of percutaneous transluminal angioplasty (PTA) due to AVF stenosis or occlusion.

On physical examination, the brachial artery pulse was diminished in patients 1–7. AVF thrill was discontinuous in patients 1–3 and 5–6, and completely absent in patients 4 and 7 due to AVF occlusion. A blowing murmur over the SA was audible in patient 2. In contrast, brachial artery pulses were intact and AVF examination was unremarkable in patients 8 and 9. Blood pressure (BP) was measured bilaterally in all patients, and systolic pressure was consistently lower on the fistula side. The median systolic BP difference between arms was 36 mmHg (range, 15–60 mmHg), while the median diastolic BP difference was 3 mmHg (range, −11 to 11 mmHg).

All patients underwent duplex ultrasound (DU) to evaluate AVF function before and after treatment. AVF blood flow was also measured using DU in a straight, stenosis-free segment of the brachial artery (Table 1). Radial artery stenosis was noted in patient 1, while cephalic vein stenosis was identified in patients 2–4 and 6–7. No vascular abnormalities were detected in the remaining three patients. Four patients (patients 1, 3–4, and 7) underwent simultaneous DU of the ipsilateral vertebral artery, complete flow reversal was observed in patients 1, 3, and 7, while patient 4 demonstrated systolic reversal only.

**FIGURE 1:**
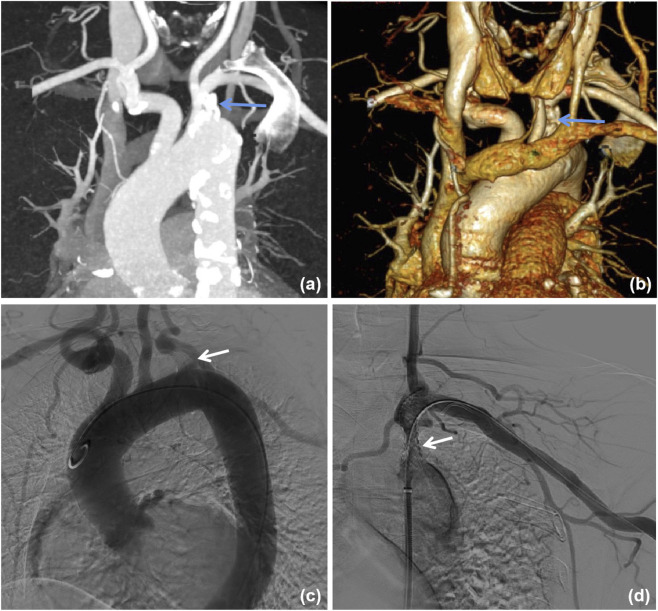
Images of subclavian artery stenosis. **(a)** Computed tomography angiography shows severe stenosis at the origin (arrow). **(b)** Stenosis (arrow) in 3D reconstruction. **(c)** Digital subtraction angiography shows occlusion at the origin of the subclavian artery (arrow). **(d)** Successful resolution of the stenosis following percutaneous transluminal angioplasty and stenting (arrow).

Both CTA and DSA were used to evaluate the severity of SA stenosis in seven patients (patients 1–7) (Figure 1). CTA revealed stenosis in patients 1, 3, and 7, whereas DSA demonstrated complete occlusion. Patients 8 and 9 underwent only CTA, which demonstrated severe stenosis of the left SA. The lesion was located at the origin of the SA in six patients (66.7%) and in the proximal segment in three patients (33.3%). The underlying etiology of SA disease was attributed to atherosclerosis in seven patients (patients 1 and 3–8, 77.8%) and to Takayasu arteritis in two patients (patients 2 and 9, 22.2%).

Details of revascularization therapy are presented in Table 1. PTA combined with intraluminal stenting was performed in patients 1–6, patient 7 underwent PTA alone, while patients 8 and 9 declined revascularization. Patients 1–4 received simultaneous PTA for coexisting AVF stenosis or occlusion. Following treatment, all patients achieved adequate AVF blood flow to support effective hemodialysis, and the reported symptoms of patients 1 and 2 were completely resolved.

Among the six patients who underwent PTA with stenting (patients 1–6), both technical success and clinical success were 100%. Follow-up outcomes are summarized in Table 1. Primary patency duration ranged from 3.2 to 34.1 months. To date, no patient has developed recurrence of SA stenosis or procedure-related complications.

## Discussion and literature review

In this case series, we described nine hemodialysis patients who developed SA stenosis or occlusion in association with dialysis access dysfunction. All patients had left-sided AVFs, with 77.8% (7/9) located at the wrist and 88.9% (8/9) being radial-cephalic fistulas. The majority presented with access-related manifestations, including reduced AVF blood flow, weak or absent thrill, AVF occlusion, or significant inter-arm blood pressure differences, while a subset also experienced neurological symptoms such as dizziness or upper limb numbness. Imaging revealed that two-thirds of lesions were located at the SA origin, with atherosclerosis being the predominant etiology, and Takayasu arteritis accounting for a smaller proportion. Most patients (seven of nine) underwent revascularization—primarily PTA combined with stenting—resulting in restoration of adequate AVF flow and symptomatic improvement, whereas two patients declined intervention and were managed conservatively.

The integrity of the vascular access circuit included the heart, arterial inflow, AVF, and venous outflow, and any disturbance within this circuit could adversely affect access flow (Lok et al., 2020). While outflow stenosis was the predominant cause of AVF dysfunction, inflow stenosis was also a critical factor, with an incidence reported as high as 35%, and therefore warranted careful attention (Asif et al., 2005; Duijm et al., 2009). Inflow stenosis could occur as an isolated lesion or in combination with outflow abnormalities (Wu et al., 2012; Caeiro et al., 2013). In our series, six of the nine patients (66.7%) had concomitant AVF stenosis or occlusion.

SA stenosis had been reported in approximately 2% of the general population and up to 7% in clinical populations (Shadman et al., 2004). It most frequently involved the proximal segment and occurred more often on the left side than on the right (Zhang et al., 2022; Bradaric et al., 2015). Established risk factors included current or prior smoking, family history, hypertension, diabetes mellitus, dyslipidemia, and peripheral arterial disease (Shadman et al., 2004; Bradaric et al., 2015). Compared with the general population, hemodialysis patients were more likely to develop peripheral vascular disease, partly due to dialysis-specific factors (O'Har et al., 2002; Raikou and Kyriaki, 2019; Krishnan et al., 2015). In one study of inflow stenosis in dysfunctional hemodialysis access, 7% of cases were attributed to subclavian artery lesions (Duijm et al., 2006). The most common etiology of SA stenosis was atherosclerosis, though inflammatory conditions (such as Takayasu arteritis and other vasculitides) and mechanical causes (including trauma and compression syndromes) were also described (Saha et al., 2017; Potter and Pinto, 2014; Furuta et al., 2015; Aboyans et al., 2018). In our series, seven patients (77.8%) had atherosclerotic lesions, while two (22.2%) had Takayasu arteritis.

Given these observations, it is important to place our findings in the context of existing literature specifically focusing on SA stenosis in hemodialysis patients with AVFs. Compared with the general population, these patients not only share common risk factors for SA disease but also face unique hemodynamic challenges imposed by the presence of high-flow AVFs. To further illustrate the spectrum of etiologies, clinical manifestations, and management approaches, we reviewed previously reported cases of SA stenosis or occlusion in this population. The key characteristics and outcomes from these reports, along with the findings from our own series, are summarized in Table 2.

**TABLE 2:** Summary of reported cases of subclavian artery stenosis in hemodialysis patients.

Reference	Patient background	Clinical features	Intervention	Outcome
Tezuka et al. (2024)	Hemodialysis patient with masked left SA stenosis after AVF creation	Access dysfunction, unequal BP between arms	PTA + stenting	AVF function restored
Agarwal et al. (2018)	Dialysis fistula with proximal SA stenosis	Subclavian steal syndrome	Stenting	Vertebral flow normalized, symptoms relieved
Tanaka et al. (2013)	Post-CABG patient with left AVF and ipsilateral SA stenosis	Coronary-subclavian steal, angina	Stenting	Coronary ischemia improved
Aihara et al. (2019)	Hemodialysis patient with recurrent AVG thrombosis due to SA stenosis	Coolness of the left upper arm and fingers, unequal BP between arms	Stenting	Blood pressure gap decreased, no recurrence of the AVG thrombosis
Sag et al. (2016)	Hemodialysis patient with left SA occlusion + severe stenosis of the right vertebral artery	Retrosternal angina happening during hemodialysis	PTA + stenting	Cardiac symptoms relieved
Lee et al. (2004)	Hemodialysis patient with ipsilateral SA stenosis	Angina and dizziness during hemodialysis	Stenting	Symptoms resolved
Clark et al. (2012)	Pontine infarction caused by subclavian steal phenomenon due to SA stenosis and an arteriovenous shunt	Transient ischemic attack symptoms with dysrhythmia during hemodialysis	PTA + stenting	Subclavian steal phenomenon no longer occurred
Hashimoto et al. (2023)	Hemodialysis patient with severe stenosis and calcified lesions in the left subclavian artery	Dizziness and pain in the left hand during hemodialysis	Common carotid-axillary bypass	Subclavian steal syndrome relieved
Kuo et al. (2010)	Hemodialysis patient with occult SA stenosis	Progressive decline of vascular access blood flow	PTA + stenting	AVF function restored
Tan et al. (2013)	Hemodialysis patient with SA stenosis and a history of quadruple coronary bypass surgery	Coronary-subclavian steal	Stenting	Continued to have episodes of monophasic ventricular tachycardia during dialysis, subsequently converted to peritoneal dialysis
Crowley et al. (2002)	Hemodialysis patient with a CABG history presented with SA stenosis	Angina, lateral chest wall pain during dialysis, and distal hypoperfusion of the left hand	Stenting	All symptoms resolved
Nanda et al. (2009)	Hemodialysis patient with CABG and SA stenosis	Three episodes of pulmonary edema within 1 h of a hemodialysis session	PTA + stenting	Dialysis without event, SA keeps patent
This case series	Nine hemodialysis patients with left SA stenosis	Dizziness, numbness in the left upper limb, decreased AVF blood flow, unequal BP between armsetc.	6 PTA + stenting; 1 PTA alone; the other two refused treatment	All treated patients restored adequate AVF flow, symptoms relieved

Abbreviations: SA, subclavian artery; AVF, arteriovenous fistula; PTA, percutaneous transluminal angioplasty.

CABG, coronary artery bypass graft surgery; AVG, arteriovenous graft.

In hemodialysis patients, SA stenosis or occlusion was an underrecognized but clinically significant cause of vascular access dysfunction. Atherosclerosis remained the predominant etiology, reported in most cases across the literature (Alemzadeh-Ansari et al., 2021; Tezuka et al., 2024; Agarwal et al., 2018; Tanaka et al., 2013; Aihara et al., 2019). Contributing risk factors included long-standing hypertension, diabetes, dyslipidemia, smoking, and the presence of peripheral arterial disease (Kargiotis et al., 2016; Kuo et al., 2010; Fields and Lemak, 1972). Inflammatory vasculopathies, such as Takayasu arteritis, though less common, had also been reported (Sag et al., 2016; Hashimoto et al., 2023). The creation of a high-flow AVF itself often exacerbated hemodynamic compromise, unmasking pre-existing subclavian lesions or aggravating latent narrowing (Alemzadeh-Ansari et al., 2021; Tezuka et al., 2024).

The clinical manifestations of SA stenosis or occlusion in hemodialysis patients primarily included vascular access–related dysfunction and subclavian steal syndrome (Table 3) (Kuo et al., 2010; Tan et al., 2013; Fields and Lemak, 1972; Kokkosis et al., 2014; Crowley et al., 2002). Access-related dysfunction typically presented as decreased AVF blood flow, weak or absent thrill, or recurrent arteriovenous graft thrombosis (Kuo et al., 2010; Kokkosis et al., 2014). In our case series, six of nine patients (66.7%) had concomitant AVF stenosis or occlusion, underscoring the high prevalence of combined inflow and outflow pathology. Subclavian steal syndrome, characterized by dizziness, visual disturbance, or focal neurological deficits, was most often associated with SA or innominate artery occlusion (Tan et al., 2013; Fields and Lemak, 1972; Crowley et al., 2002). However, several reports showed that steal phenomena could also occur in patients with AVFs even in the absence of arterial stenosis, due to the hemodynamic burden of high AVF flow (Alemzadeh-Ansari et al., 2021; Kaneko et al., 2018). Patients with dialysis access were more likely than nondialysis patients to demonstrate retrograde vertebral artery flow, a key hemodynamic sign of steal (Naidich et al., 2018). Interestingly, while most published reports of SA stenosis in dialysis patients emphasized steal-related symptoms as the main presentation, our cohort predominantly presented with vascular access dysfunction, which might have reflected referral bias since neurological complaints were often managed in non-access settings.

**TABLE 3:** The clinical manifestations of hemodialysis patients with SA stenosis or occlusion.

Subclavian steal syndrome	Vascular access related performance
Mostly asymptomatic Vertebrobasilar insufficiency Ipsilateral upper extremity ischemia Myocardial ischemia in CABG patients with internal mammary artery	May be asymptomatic Insufficient hemodialysis blood flow Abnormal physical examination of access may include weakened and/or discontinuous fistular thrill, decreased brachial artery pulsation, poor pulse augmentation test and unequal blood pressure of both upper limbs

Abbreviations: CABG, coronary artery bypass graft surgery.

Endovascular therapy remained the mainstay of treatment. PTA with stenting consistently demonstrated high technical success and durable outcomes, with resolution of ischemic symptoms and restoration of adequate AVF flow (Tan et al., 2013; Alemzadeh-Ansari et al., 2021; Tezuka et al., 2024; Agarwal et al., 2018; Tanaka et al., 2013; Aihara et al., 2019; Sag et al., 2016; Lee et al., 2004; Schoenkerm et al., 2009; Nanda et al., 2009). For example, Agarwal et al. (2018) reported normalization of vertebral flow and relief of dizziness, while (Nanda et al., 2009) described resolution of recurrent pulmonary edema after subclavian stenting. In anatomically complex cases or when endovascular management was not feasible, surgical bypass procedures such as carotid-axillary bypass had been reported with favorable outcomes (Hashimoto et al., 2023). Flow modulation procedures were occasionally considered when symptoms were primarily due to high AVF flow with borderline SA lesions, although this was less common when anatomic stenosis was confirmed. Overall, conservative management rarely sufficed once dialysis adequacy or myocardial/cerebral perfusion was compromised.

**FIGURE 2:**
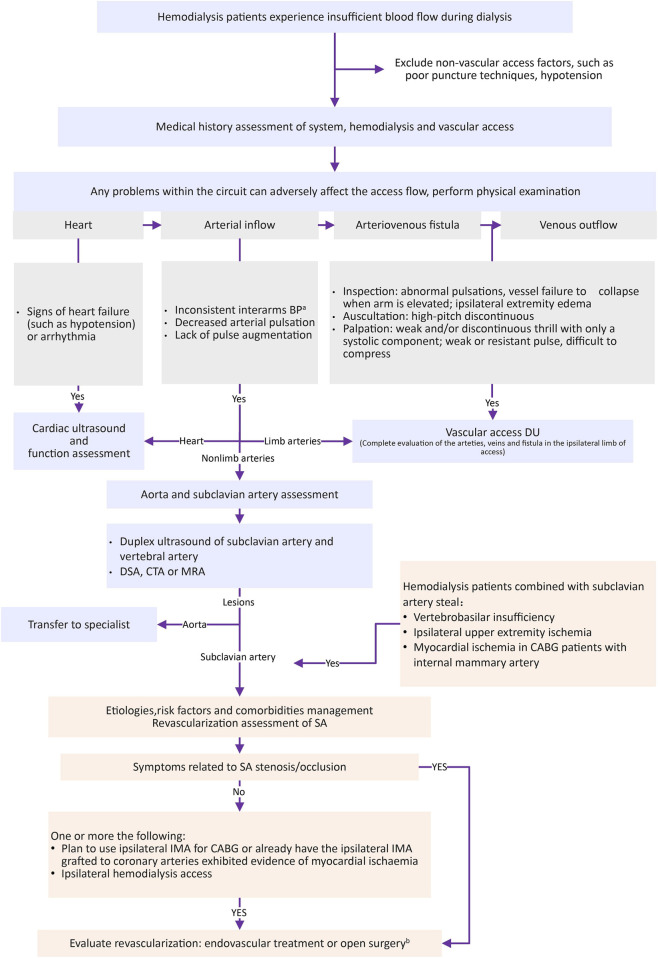
Identify and manage SA stenosis in hemodialysis patients with complaints of insufficient blood flow. Abbreviations: BP, blood pressure; CABG, coronary artery bypass graft surgery; CTA, computed tomography angiography; DSA, digital subtraction angiography; DU, duplex ultrasound; HD, hemodialysis; IMA, internal mammary artery; MRA, magnetic resonance angiography; PAD, peripheral artery disease; SA, subclavian artery. a: The interarm difference in systolic blood pressure exceeds 10 mmHg. b: Endovascular treatment is often the default strategy.

Building on the findings from our case series and the broader literature, we recognized the need for a practical approach to guide the evaluation and management of hemodialysis patients with suspected SA stenosis. To this end, we outline a practical clinical pathway (Figure 2) designed to facilitate early detection, accurate diagnosis, and timely intervention. This protocol emphasizes systematic screening, integration of imaging modalities, and individualized treatment decisions to optimize dialysis adequacy and reduce morbidity.

As shown in Figure 2, while screening for heart, AVF and venous outflow, an understanding of how to identify SA stenosis, thorough clinical history and physical examination are key steps for the early detection of SA stenosis (Aboyans et al., 2018). Comparing blood pressures in both arms and checking upstream arterial pulses are irreplaceable and recommended as first steps (Kuo et al., 2010; Saha et al., 2017), and further vascular assessment should be conducted if the difference in systolic blood pressure exceeds 10 mmHg (Clark et al., 2012). SA stenosis should be highly suspected when patients have subclavian artery steal (Kargiotis et al., 2016; Saha et al., 2017). DU is often a first step in the vascular workup both for screening and diagnosis, Doppler assessment of SA enables the detection of high-velocity flows indicating >50% stenosis (Kalaria et al., 2005; Aboyans et al., 2017), and reversed blood flow in the hemodialysis access ipsilateral vertebral artery also indicates SA stenosis (Saha et al., 2017; Aboyans et al., 2018; Bron et al., 2010). CTA and magnetic resonance angiography are reliable inspection methods for SA stenosis, and they can also provide extravascular information other than vascular information (Aboyans et al., 2018). DSA is considered the standard reference in the vasculature and can be combined with endovascular therapy (Aboyans et al., 2018). SA stenosis should also be considered when there is suboptimal tension, weak thrill, and/or sluggish blood flow on postintervention angiogram or inadequate blood flow at the first dialysis session after AVF intervention (Wu et al., 2012).

To reduce mortality, improve symptoms, and ensure normal dialysis in hemodialysis patients with SA stenosis, in addition to the management of etiologies, risk factors and comorbidities, it is necessary to evaluate the revascularization of SA (Aboyans et al., 2018) (Figure 2) and to treat AVF lesions if AVF disease is present (Kuo et al., 2010). Revascularization of SA includes endovascular treatment and open surgery, and the former is often the default strategy (Aboyans et al., 2018). A systematic review (544 patients) revealed that stenting after angioplasty for SA stenosis and maintenance of patency at 1 year is superior to angioplasty alone (Chatterjee et al., 2013), but there are no randomized controlled trials to determine. There are few reports on revascularization for asymptomatic patients with sufficient AVF blood flow. Two (patients 8 and 9) in our study did not receive endovascular treatment or surgery for SA stenosis, and they could continue dialysis normally. Based on our experience and the available literature, stent placement may be considered in hemodialysis patients who exhibit access dysfunction and SA stenosis or occlusion, although high-quality evidence comparing stenting with angioplasty alone remains limited. This intervention appears to improve access flow and may reduce the risk of recurrent dysfunction related to inflow stenosis.

This study has limitations inherent to the case series. The sample size was small and from a single center, limiting generalizability. Follow-up duration was limited and variable among patients. No formal statistical analyses or comparative assessments were performed. Therefore, our findings are descriptive and practice-oriented, and warrant further validation in larger studies.

## Conclusion

SA stenosis is a critical but often overlooked cause of access dysfunction in hemodialysis patients. In our case series, timely diagnosis and stent-based revascularization restored AVF flow and improved clinical outcomes. Literature review confirms that high-flow AVFs and atherosclerosis increase SA stenosis risk. Early screening with bilateral blood pressure measurement and duplex ultrasound is essential. Stent-based revascularization should be considered in patients with SA-related access dysfunction. Developing a practical diagnostic and treatment approach may help improve dialysis adequacy and reduce the risk of hemodialysis failure in this high-risk group.

## Data Availability

The raw data supporting the conclusions of this article will be made available by the authors, without undue reservation.
